# An Ethyl Methanesulfonate-Induced *GIF1* Splicing Site Mutation in Sesame Is Associated with Floral Malformation and Small Seed Size

**DOI:** 10.3390/plants13233294

**Published:** 2024-11-23

**Authors:** Guiting Li, Hengchun Cao, Qin Ma, Ming Ju, Huili Wang, Qiuzhen Tian, Xiaoxu Feng, Xintong Zhang, Jingjing Kong, Haiyang Zhang, Hongmei Miao

**Affiliations:** 1 Henan Sesame Research Center, Henan Academy of Agricultural Sciences, Zhengzhou 450002, China; liguiting07111106@163.com (G.L.); chczhima@163.com (H.C.); maqin073130@163.com (Q.M.); jumingzz@163.com (M.J.); whl800222@163.com (H.W.); tianqiuzhen@163.com (Q.T.); bbxe2013@163.com (X.F.); ZT921205@163.com (X.Z.); kongjingj@163.com (J.K.); 2 Key Laboratory of Specific Oilseed Crops Genomics of Henan Province (Henan Sesame Research Center, Henan Academy of Agricultural Sciences), Zhengzhou 450002, China

**Keywords:** sesame, flower development, seed size, genome-wide association study, *GIF1*

## Abstract

Flower and inflorescence architecture play fundamental roles in crop seed formation and final yield. Sesame is an ancient oilseed crop. Exploring the genetic mechanisms of inflorescence architecture and developmental characteristics is necessary for high-yield breeding improvements for sesame and other crops. In this study, we performed a genetic analysis of the sesame mutant *css1* with a malformed corolla and small seed size that was mutagenized by ethyl methanesulfonate (EMS) from the cultivar Yuzhi 11. Inheritance analysis of the cross derived from *css1* mutant × Yuzhi 11 indicated that the mutant traits were controlled by a single recessive gene. Based on the genome resequencing of 48 F_2_ individuals and a genome-wide association study, we determined SNP9_15914090 with the lowest *p* value was associated with the split corolla and small seed size traits, which target gene *Sigif1* (*GRF-Interacting Factor 1*)*. SiGIF1* contains four exons and encodes a coactivating transcription factor. Compared to the wild-type allelic gene *SiGIF1*, *Sigif1* in the mutant *css1* has a splice donor variant at the exon2 and intron2 junction, which results in incorrect transcript splicing with a 13 bp deletion in exon2. The expression profile indicated that *SiGIF1* was highly expressed in the flower, ovary, and capsule but lowly expressed in the root, stem, and leaf tissues of the control. In summary, we identified a gene, *SiGIF1*, that regulates flower organs and seed size in sesame, which provides a molecular and genetic foundation for the high-yield breeding of sesame and other crops.

## 1. Introduction

Sesame (*Sesamum indicum* L., 2*n* = 26) belongs to the *Sesamum* genus of the Pedaliaceae family and is an ancient oilseed crop. Currently, sesame is widely cultivated in tropical and subtropical regions because of its high tolerance to high temperatures and arid environments [1]. Sesame seeds are a rich source of nutrients. As health consciousness has increased, the demand for sesame has increased. Thus, breeding varieties with increased yield potential remains the most important objective for sesame cultivation.

Understanding the physiological and molecular mechanisms of flower and seed development in sesame is crucial for enhancing theoretical knowledge and practical breeding techniques. Sesame flowers exhibit indeterminate inflorescence, with one or more (typically three) flowers per leaf axil [2]. Research has indicated that functional deficiency of *centroradialis-like* (*CEN2*) results in three flowers per leaf axil, highlighting its role in flower number determination in sesame [3,4]. The floral development process comprises inflorescence primordia, brace primordia, and terminal floral primordia [5]. However, floral malformations caused by phytoplasma infection can disrupt this process, either by suppressing floral development-related genes or proteins, and inducing negative regulators or repressors via comparative proteome and transcriptome analyses [6,7]. Despite this, the molecular genetic information underlying normal floral developmental traits in sesame remains largely unexplored, and no genes have been cloned.

In *Arabidopsis*, floral organ development has been well studied and occurs in three stages. The shoot apical meristem develops into the inflorescence meristem, which then develops as the floral meristem (FM). The FM produces different types of functional flowers [8,9,10]. Many genes involved in this process have been identified [11,12,13], e.g., MONOPTEROS, an auxin-responsive transcription factor crucial for FM development. Mutants without MONOPTEROS function produce naked inflorescence “pins” devoid of flowers [12]. Another key regulator is AGAMOUS, which controls FM activity. Mutants without AGAMOUS function exhibit transformations in two adjacent whorls of flowers [11,13].

EMS alkylates the guanine base, with G/C pairing base transitions by any other base, and EMS mutant libraries are powerful tools to identify genes linked to key traits in plants. In a sesame EMS mutant library created by scientists in China (with Yuzhi 11 selected as the EMS mutagenesis material) [14], we found a *css1* mutant exhibiting a split corolla that exposed the stamen and pistil, along with small mature seeds, contrasting with the sesame cultivar Yuzhi 11. This mutant was deemed valuable for exploring the molecular mechanisms of floral and seed development in sesame.

Thus, in this study, we systematically investigated the morphological characteristics and genetic background of the *css1* mutant. Using inheritance analysis and a genome-wide association study (GWAS) of variant information from the F_2_ population, we identified the *GIF1* (*GRF-Interacting Factor 1*) gene associated with floral and seed development. Research on *Arabidopsis thaliana* has shown that the *GIF1* gene positively regulates leaf, petal, and seed development by influencing cell number and size [15,16]. Similarly, in rice, *OsGIF1* acts as a transcriptional cofactor, positively regulating organ size by modulating cell expansion [17]. Finally, we explored the gene expression and evolutionary characteristics of *SiGIF1* in Yuzhi 11. These findings provide a theoretical basis for understanding floral development and enhancing yield in sesame.

## 2. Result

### 2.1. Phenotype and Inheritance Analyses

**Figure 1:**
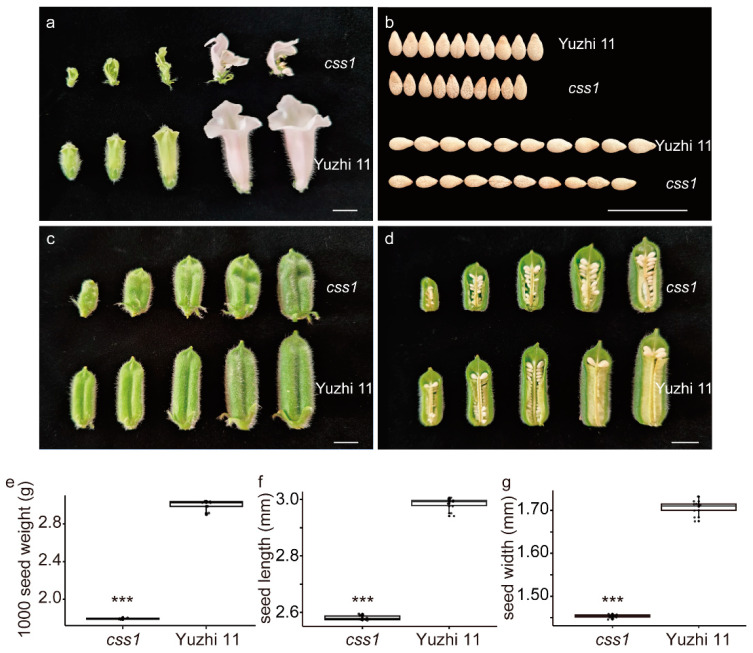
Morphological comparisons in corolla (**a**), seed size (**b**), and capsule (**c**,**d**) between Yuzhi 11 and mutant *css1*. Scale bar = 1 cm. (**e**–**g**) Thousand-seed weight, seed length, and seed width of plants in (**b**). *p* values from *t* tests are shown (*** *p* < 0.001). All boxplots show the upper and lower quartiles separated by the median (horizontal line). Whiskers represent maximum and minimum values.

Phenotypic observations showed significant differences in morphology of flower, capsule, and seed size between the *css1* mutant and Yuzhi 11 (wild-type parent). In particular, during the bloom period, the mutant exhibits a split-like corolla and a concave pericarp (Figure 1a,c,d). Additionally, the mature seed size of the *css1* mutant was significantly smaller than that of Yuzhi 11 (Figure 1b,e–g), with a thousand-seed weight of approximately 1.80 g (the thousand-seed weight of Yuzhi 11 was approximately 2.99 g).

To clarify the inheritance of the mutant phenotype, we constructed F_1_ hybrids and F_2_ populations from cross-combinations of mutant *css1* and Yuzhi 11. All F_1_ generations demonstrated a wild-type phenotype similar to that of Yuzhi 11. Of the 384 F_2_ individuals, 298 and 86 had wild-type and mutant phenotypes, respectively, which were separated in an expected ratio of 3:1. Additionally, we calculated the chi-squared tests by using the “chisq.test” function in R for phenotypic segregation, resulting in λ^2^ and *p* values of 0.58328 and 0.445, respectively. These results demonstrated that the phenotypic segregation in the F_2_ population fitted the Mendelian inheritance mode and that the mutant traits were controlled by a single recessive gene.

### 2.2. GWAS Analysis of F_2_ Population for Candidate Intervals

To accurately identify the candidate interval for the mutant phenotype, we resequenced 19 recessive homozygous individuals (mutant phenotype) and 29 dominant homozygous individuals (WT phenotype) from the F_2_ population, whose genotype was identified based on F_2:3_ families produced by strict self-pollination. In total, 327.4 Gb of clean data were obtained, with an average genome coverage of 22-fold per sample (Supplementary Table S2). These data were mapped to the Yuzhi 11 reference genome, and 373,257 high-quality single-nucleotide polymorphisms (SNPs) and 195,098 high-quality insert–deletion (InDel) variants were identified.

**Figure 2:**
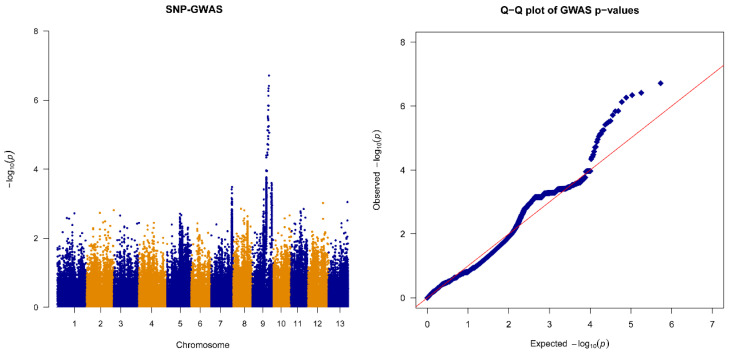
Manhattan plot and quantile–quantile plot of SNP association mapping. Each dot represents an SNP variant. Detailed information on significant loci for all traits is listed in Supplementary Table S3.

**Figure 3:**
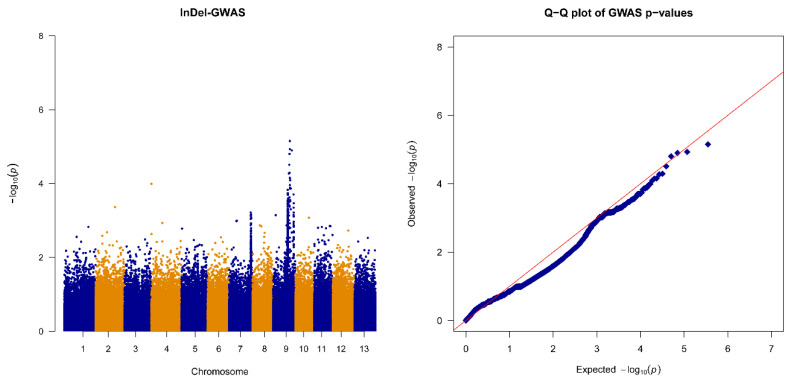
Manhattan plot and quantile–quantile plot of InDel association mapping. Each dot represents an InDel variant. Detailed information on significant loci for all traits is listed in Supplementary Table S4.

Subsequently, a GWAS with a generalized linear model was performed separately for the SNPs and InDels. For SNP association mapping, we found 26 variant sites with *p* values less than 1 × 10^−5^ located at 13,293,357–16,070,627 bp on Chr.9 (Figure 2 and Supplementary Table S3). Similarly, InDel association analysis identified 10 variant sites located at 14,178,896–16,876,339 bp on Chr.9 (Figure 3 and Supplementary Table S4). The overlapping correlation interval indicated the reliability of the results. The candidate region was thus determined to be located at 14,178,896–16,070,627 bp on Chr.9.

### 2.3. Identification of GIF1 Gene by Using Large Sets of Germplasm and Functional Annotation

Because the causal variant loci leading to mutant phenotype should not be present in natural resources with normal phenotype, we filtered the 36 detected SNP/InDel variants by using the regional genome variant data of the 560 sesame germplasm accessions. The screening results showed that five SNP variants and two InDel variants existed only in *css1* mutant within the target interval (Table 1, Table S3, and Table S4). Further functional annotation showed that the variant locus C9_15914090, with the lowest *p* value, was a splice donor variant and might result in an error in *SiGIF1* splicing of the second intron. The remaining variant loci were located in the intron, upstream, and intergenic regions, which are highly unlikely to cause a loss in gene function. Therefore, the splice donor site mutation (C9_15914090, G→A) was considered the candidate mutant locus for the *css1* mutant.

**Figure 4:**
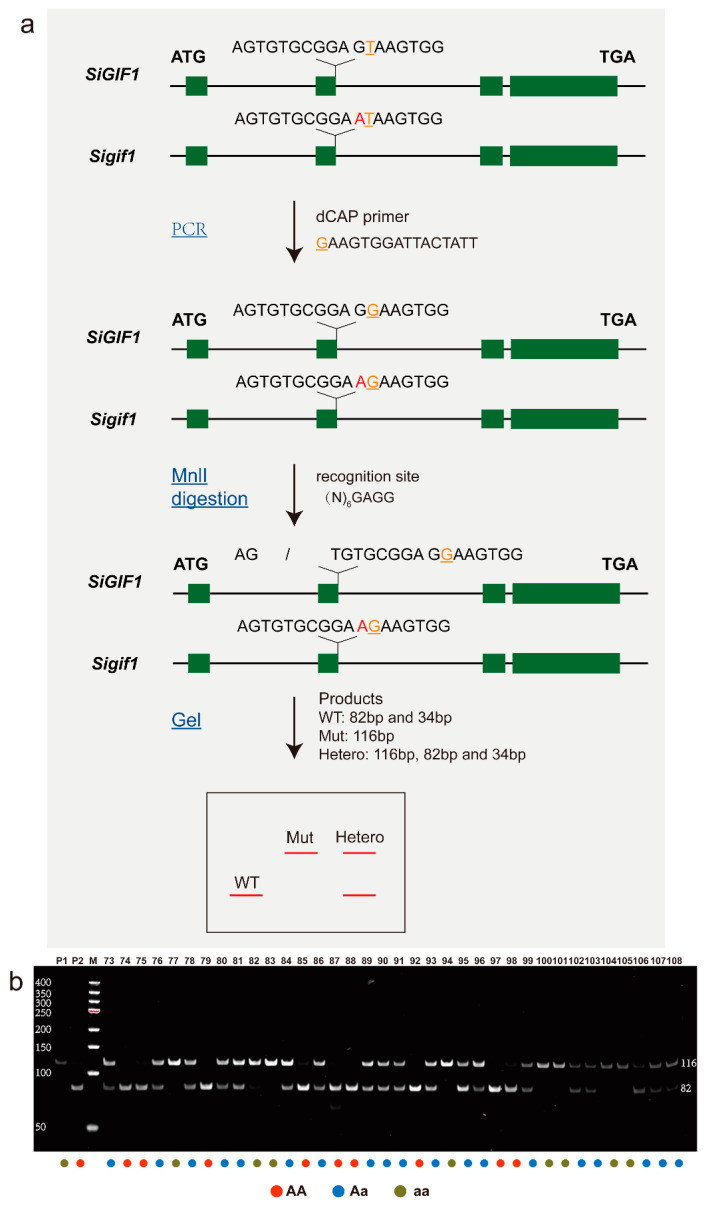
F_2_ population validation of C9_15914090 SNP by MnII dCAPS assay. (**a**) Theory of the MnII dCAPS assay, including creating the MnII recognition sites and discerning the wild and mutant sequences. Red bases indicate C9_15914090 mutant SNP. Orange bases indicate the artificially created MnII recognition site. (**b**) Partial population validation results (samples 73–108) using 10% nucleic acid PAGE. P1, parent with recessive homozygous genotype; P2, parent with dominant homozygous genotype. Numbers on the left and the right of the image represent the molecular sizes of the marker and PCR product, respectively. Full results (genotypes and phenotypes) are listed in Supplementary Table S5.

We then used dCAPS assays to validate this putative mutant locus in additional F_2_ individuals, including 70 individuals with dominant homozygous genotypes, 60 individuals with recessive homozygous genotypes, and 140 individuals with heterozygous genotypes. As shown in Figure 4a, we first created MnII recognition sites via a mismatch in the PCR primers for each F_2_ individual. Next, we analyzed the MnII-digested PCR products based on 10% nucleic acid PAGE and observed whether the actual genotypes were consistent with the genotypes judged based on the F_2:3_ family. The results showed that the C9_15914090 alleles correlated entirely with the phenotype in the test population (Figure 4b and Supplementary Table S5); therefore, the *SiGIF1* gene containing the C9_15914090 locus was regarded as a candidate gene.

### 2.4. Incorrect Splicing Event Causes Loss of SiGIF1 Function

**Figure 5:**
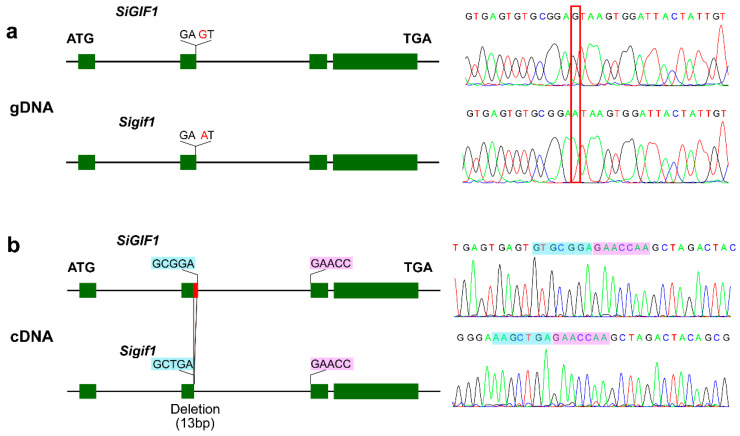
Mutant loci comparison of Yuzhi 11 and *css1* mutant in gDNA and cDNA. (**a**) At the genome level, the causal SNP (G to A) is located on the splice_donor_region of the second exon for the *SiGIF1* gene. Red font (left) and red boxes (right) indicate the position of C9_15914090. (**b**) At the transcription level, the 13 bp deletion on the second exon (red solid box) was induced in the *css1* mutant. Bases with blue and purple backgrounds indicate connecting bases of second exon and third exon for Yuzhi 11 (up) and *css1* mutant (down), respectively.

The *SiGIF1* gene encodes transcriptional coactivators of plant-specific growth-regulating factors (GRFs) and plays a crucial role in plant developmental properties [15,16,18]. Gene structure analysis showed that the *SiGIF1* gene consisted of four exons and three introns and that the C9_15914090 splice_donor_alternative site occurred at the exon2 and intron2 junction (Figure 5a). To verify that this mutant locus causes errors in splicing in exon2, we designed primer pairs to amplify the entire cDNA sequence of the *css1* mutant. Notably, we found a 13 bp deletion in exon2 at the transcript level (Figure 5b). These results indicated that the G to A mutation eliminates a canonical donor splicing site in intron2 and a cryptic site in exon2, located 13 nucleotides upstream of the mutation, and is used as a donor site. As a result, 13 bp are deleted from exon2 transcript, introducing a frameshift in the mRNA translation and a premature termination codon in the protein. The protein sequence of SiGIF1 and Sigif1 are shown in Supplementary Table S6. Thus, we posited that *SiGIF1* is the causal gene of the *css1* mutant.

### 2.5. SiGIF1 Encodes Nucleus-Localized Transcriptional Coactivators

**Figure 6:**
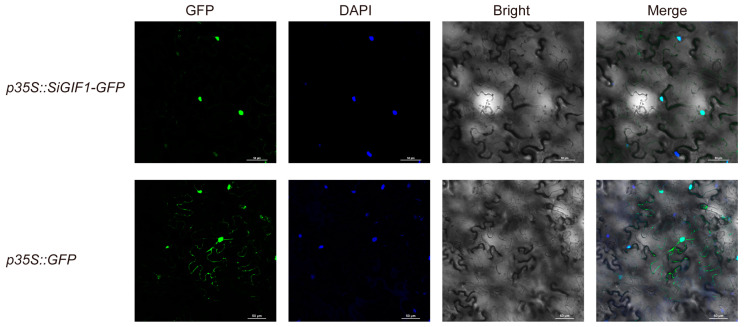
Subcellular localization of SiGIF1 protein. Confocal images of tobacco leaves after 72 h of infection. Scale bar = 50 μm.

To investigate the subcellular localization of SiGIF1, we transformed *p35S:: SiGIF1-GFP* into *Agrobacterium tumefaciens* GV3101. They were then inoculated into *Nicotiana benthamiana* leaves, with GFP expression observed under a confocal microscope (Figure 6). The results showed that SiGIF1 was mainly expressed in the nucleus, which is consistent with findings of an existing study that GIF proteins function as transcriptional coactivators by forming complexes with GRF transcription factors. However, the NLS sequence was not identified in SiGIF1 through online prediction tool (https://www.novoprolabs.com/tools/nls-signal-prediction, accessed on 22 September 2024).

### 2.6. High Expression of SiGIF1 Gene in Flower, Ovary, Pericarp, and Seed

**Figure 7:**
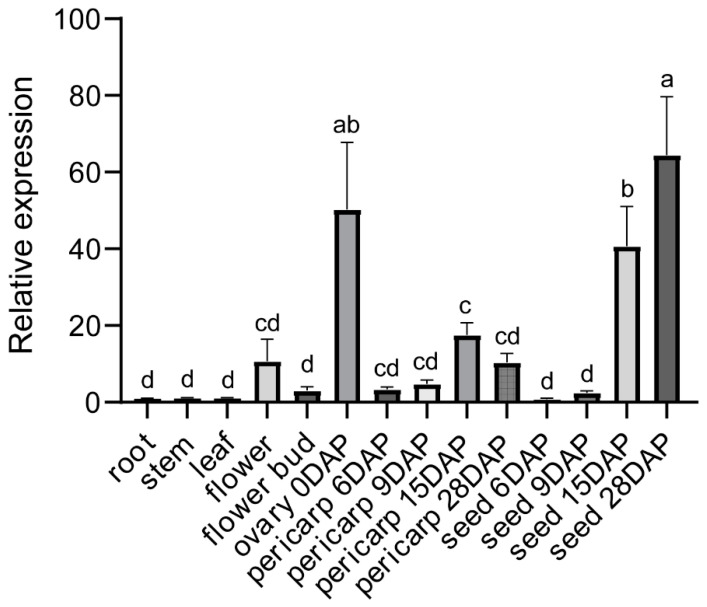
Expression profile of *SiGIF1* in different tissues of Yuzhi 11 using RT-qPCR. The endogenous *Siβ-tubulin* gene was used to normalize the expression level. Statistical analysis was performed by one-way ANOVA analysis with Dunnett’s multiple comparisons test, and different lower-case letters above columns indicate statistical differences at *p* < 0.05; data are provided as means ± SDs. Young root, stem, leaf, bud, flower, ovary, pericarp, and seed tissues from 6 to 28 DAP are assayed. DAP, days after pollination. The darker the bar graph, the higher the relative level of expression.

To acknowledge the importance of the *SiGIF1* gene in the flower and seed development of sesame, we detected the expression pattern of the *SiGIF1* gene in different tissues of Yuzhi 11 using RT-qPCR, finding that *SiGIF1* expression was high in the flower and ovary tissues and low in the root, stem, and leaf tissues (Figure 7). Furthermore, we found that the expression was gradually upregulated during pericarp and seed development (Figure 7), suggesting that *SiGIF1* gene is involved in the capsule and seed development in sesame.

### 2.7. Evolutionary Conservation of GIF1 Gene in Sesamum

**Figure 8:**
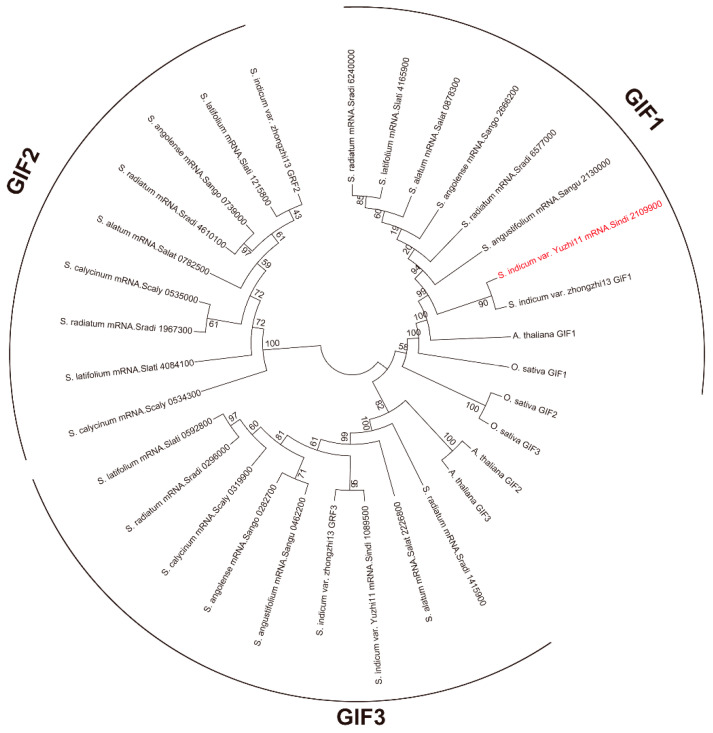
Phylogenetic relationship of *GIF1* genes and its homologs in *Arabidopsis*, rice, and two cultivated sesames (*S. indicum* var. Yuzhi 11, *S*. *indicum* var. zhongzhi 13) and five ancestral sesames (*S. alatum*, *S. latifolium*, *S. angolense*, *S. angustifolium*, *S. radiatum*). Red font represents *S. indicum* var. Yuzhi11. The numbers on the branches represent bootstrap values.

Furthermore, we searched all *GIF* gene families in *Arabidopsis*, rice, and two cultivated (*Sesamum indicum* var. Yuzhi11, and *S*. *indicum* var. zhongzhi13) and five ancestral sesames (*S. alatum*, *S. latifolium*, *S. angolense*, *S. angustifolium*, and *S. radiatum*) by using sequence alignment and phylogenetic relationship construction (Figure 8 and Figure S1). The results showed that the *GIF1* gene was retained in all seven sesame species, including diploid sesame with one sequence and tetraploid sesame with two sequences owing to hetero-tetraploid tetraploidy (*S. radiatum*). Furthermore, we found that *Arabidopsis*, rice, and sesame *GIF1* genes clustered in the same clade and were separated from *GIF2* and *GIF3*. And the *GIF1* gene of Yuzhi 11 shows 49.6% identity and 58.5% similarity with rice, and 65.5% identity and 70.5% similarity with *Arabidopsis* calculated by EMBOSS Water (https://www.ebi.ac.uk/, accessed on 20 September 2024). These results indicated that the *GIF1* gene might be present in early angiosperms and retained in sesame species without loss or expansion, suggesting that the gene might be functionally conserved in plants.

## 3. Discussion

### 3.1. Rapid Identification of EMS-Induced Causal Mutation via Mapping-by-Sequencing

The experimental process for identifying EMS-induced causal genes, involving crossing the mutants with the wild-type, followed by whole-genome resequencing and linkage analyses, was published in *Nature Protocols* in 2016 [19]. Notably, in the genetics field, linkage analysis of segregating populations remains a classical, reliable method for the fine mapping of functional genes, which has been confirmed in many studies. For instance, a recent study published in a leading botany journal demonstrated the successful use of linkage analysis of F_2_ segregating populations to identify two genes responsible for the EMS mutant with yellow leaf trait in Chinese cabbage [20]. Similarly, another influential journal in genetics reported the application of linkage analysis in gene mapping involved in the short fiber phenotype in cotton from an EMS mutant [21]. In this study, a GWAS of the F_2_ segregating population was performed (Figure 2 and Figure 3), identifying 36 candidate variant loci. After filtering based on larger sets of germplasm and functional annotation, only the splice donor site mutation (C9_15914090, G→A) of *SiGIF1* was retained, with mutation characteristics aligning with EMS-alkylated G/C. Thus, the variant locus C9_15914090 was considered the causal mutant locus for the *css1* mutant. A similar approach has been successfully applied in cloning multiple genes related to key agricultural traits in sesame, such as capsule length, internode length, and non-shattering phenotype, induced by EMS [22,23,24].

### 3.2. Pleiotropic Effects of SiGIF1 on Sesame Development

GIF1, one of the transcriptional coactivators of plant-specific GRFs, has been characterized in *Arabidopsis*, rice, maize, wheat, pear, *Phalaenopsis equestris*, and other plants [15,17,25,26,27,28,29]. In *Arabidopsis*, GIF1 acts as a positive regulator of organ size by regulating cell proliferation, as shown especially by the *gif1* mutant that developed narrow leaves and petals with few cell numbers, which was correlated with small shoot apical meristem and leaf primordia [15,16,18]. AtGIF1 also plays a positive role in seed size control, owing to increased cell proliferation in integuments [16]. A study on the *GIF* gene family found that the *gif1 gif2 gif3* triple mutant did not establish normal gynoecia and embryo [30]. In maize, loss-of-function *gif1* mutants have uncontrolled indeterminacy in shoot meristems, fasciated inflorescence meristems, and indeterminate axillary meristems, resulting in narrow leaves, short internodes, and highly branched ears [25,26]. In rice, *OsGIF1* positively regulates grain size and yield [17,31,32]. Similar phenotypes have been observed in other plants [17,27,29]. In this study, we confirmed that the *SiGIF1* gene is orthologous to *AtGIF1* and *OsGIF1* via homologous gene alignment and phylogenetic relationship analysis (Figure 8). We also observed that the loss-of-function *gif1* in sesame demonstrated similar phenotypes in terms of organ size and morphological changes, including a split corolla and small seed size (Figure 1). The *SiGIF1* gene was consistently expressed at high levels in the flower, ovary, pericarp, and seed development (Figure 7), further confirming its important and pleiotropic role in the development of sesame.

### 3.3. Regulatory Network of GIF1 Gene

Although our study did not demonstrate the *SiGIF1* regulatory network in sesame, this network has been extensively studied in other plants. Regarding the upstream regulated genes of *GIF1*, studies in pear have found that the RR1 (two-component response regulator) protein binds to the promoter of *PbGIF1* and activates its transcription [29]. In *Arabidopsis*, KIX8/9 (kinase-inducible domain interacting 8/9) and PPD1/2 (peapod 1/2) interact with MYC3/4 (myelocytomatosis 3/4) to form the KIX-PPD-MYC complex, which binds to the G-box sequence in the promoter of *GIF1* and represses its expression [16]. Many studies have focused on GIF1 as a transcriptional coactivator that binds to plant-specific GRFs to form the GRF1-GIF1 complex in the nucleus. However, recent studies have also identified additional proteins involved in complex assembly. For example, in maize, the GIF1-GRF1 complex recruits SWI/SNF chromatin-remodeling ATPases to influence DNA accessibility in the promoter regions of target genes associated with hormone biosynthesis, meristem identity, and determinacy [26]. In *Arabidopsis*, SOD7 and DPA4, which are B3 transcriptional repressors with functional redundancy, repress the interaction between GRF1 and GIF1. The SOD7/DPA4-GIF1-FIT module plays an important role in fine-tuning the expression of genes involved in organ size and Fe-deficiency responses [28].

### 3.4. Possible Functional Differentiation of GIF Gene Family in Sesame

In *Arabidopsis*, *GIF1* belongs to a small gene family comprising *GIF1*, *GIF2*, and *GIF3*, all of which have an overlapping function in cell proliferation and lateral organ growth [18]. In our analysis, the sesame *GIF* gene family contained three family members (*GIF1*, *GIF2*, and *GIF3*) (Figure 8). Additionally, we observed that a single mutant *gif1* (EMS mutant, Figure 1) produces large phenotypic differences; however, the phenotype of *gif2* and *gif3* remains unclear. Therefore, our further research will focus on exploring the functional redundancy and specific differentiation of the three family members by using gene expression patterns, protein location, and functional exploration.

In summary, we identified a gene, *SiGIF1*, which positively regulates floral organs and seed size in sesame. This study provides ideas for locating genes from EMS mutants and helps lay the foundation for sesame breeding with high grain yield.

## 4. Materials and Methods

### 4.1. Plant Materials

The mutant *css1* was induced from Yuzhi 11, treated with EMS, and then subjected to more than five generations of self-pollination before genetic analysis. To determine the inheritance characterization of the *css1* mutant, we made 384 F_2_ crossed individuals between the *css1* mutant and the original WT parent Yuzhi 11. All of the aforementioned germplasm and F_2_ populations were cultured at the Yuanyang experimental station (Yuanyang, China; 113°97′ E and 35°05′ N) for phenotype observation and were available from the Henan Sesame Research Center, Henan Academy of Agricultural Sciences (Zhengzhou, China).

### 4.2. Morphological Analyses

Seed length, width, and thousand-seed weight were measured using an automatic seed-size-analyzing system (SC-G, Wans hen, Hangzhou, China). The results were visualized using the R package. *p* values were obtained from Student’s *t*-test with * indicating *p* < 0.05 for significant differences, ** indicating *p* < 0.01, and *** indicating *p* < 0.001 for highly significant differences.

### 4.3. Genomic DNA and RNA Extraction

Young leaves of plant materials were collected, immersed in liquid nitrogen, and frozen at −80 °C for subsequent molecular assays. Genomic DNA and total RNA were extracted using the DNeasy Plant Mini Kit (QIAGEN, Hilden, Germany) and TRIzol reagent (Invitrogen, Shanghai, China), respectively, according to the manufacturer’s instructions and an existing study [24].

### 4.4. F_2_ Population Resequencing and Data Analyses

Forty-eight F_2_ individuals were selected for resequencing analysis. Whole-genome resequencing was performed using an Illumina HiSeq 2500 (Illumina, Inc., San Diego, CA, USA) with paired-end 150 bp reads at the Annoroad Gene Technology Corporation (Beijing, China). Clean reads were mapped to the Yuzhi 11 reference genome [33] using BWA 0.7.15 with default parameters [34]. SNPs and InDels were identified based on aligned results by GATK software (https://www.broadinstitute.org/gatk/guide/best-practices.php, accessed on 25 September 2023) and then filtered by the following parameter: --filter-expression “QD < 2.0||MQ < 40.0||FS > 60.0||SOR > 3.0||MQRankSum < −12.5||ReadPosRankSum < −8.0”. Finally, the genomic positions of SNPs and InDels were annotated using SnpEff software [35].

### 4.5. Fine Mapping of SNP/InDel Markers and Candidate Genes

To identify map markers that determine the mutant phenotype, we performed a GWAS by using the general linear model in GAPIT3 software [36] based on genome-wide SNPs and the InDel database. The Manhattan and quantile–quantile plots were visualized using the R packages “qqman” and “qq”, respectively. Furthermore, to reduce the false-positive results of the GWAS analysis, we filtered SNP/InDel variants by using the regional variant marker data of 560 sesame accessions (wild type) obtained in an existing study conducted in our laboratory [33]. Finally, we screened candidate markers and genes based on the marker mutation type and gene function.

### 4.6. PCR Analysis of the Splice Donor Mutant

Genomic DNA and cDNA templates were used to verify the splice donor mutant. The primer sequences are listed in Supplementary Table S1. cDNA was synthesized via reverse transcription using poly(A) primers. PCR conditions were as follows: initial denaturation at 94 °C for 5 min, 38 cycles of 94 °C for 30 s, 30 s at the appropriate temperature (depending on the primer set used), and 72 °C for 30 s, and an extension at 72 °C for 10 min. The PCR products were sent to TSINGKE Biological Technology (Beijing, China) for Sanger sequencing.

### 4.7. SNP Marker Verification in a Large F_2_ Population by Using Derived Cleaved Amplified Polymorphic Sequence (dCAPS) Assays

The dCAPS assay is a modification of the CAPS (or PCR-RFLP) technique used for SNP detection (https://www.ncbi.nlm.nih.gov/probe/docs/techdcaps/, accessed on 6 January 2024). In the dCAPS assay, a mismatch in the PCR primers was used to create restriction endonuclease-sensitive polymorphisms. The PCR products were detected on 10% nucleic acid PAGE. The dCAP primers used are listed in Supplementary Table S1.

### 4.8. Subcellular Localization

Subcellular localization was performed on tobacco leaves according to existing methods. First, to amplify the cDNA of *SiGIF1*, we designed the gene-specific primers SiGIF1-Sl-F and SiGIF1-Sl-R (Supplementary Table S1), each with restriction sites Kpn I and Xba I, by using Primer 3.0. Next, the cDNA of *SiGIF1* was cloned into the pCAMBIA2300-GFP vector to generate *p35S::GFP-SiGIF1*. The recombinant constructs were transformed into *Agrobacterium tumefaciens* GV3101 cells using the freeze–thaw method and cultured on LB solid medium (with Rifampicin and Kanamycin) to obtain positive transformants. Finally, a syringe injected the bacterial cells into the lower epidermis of three-week-old tobacco leaves. After 72 h, samples were collected, and fluorescence signals were detected using a confocal fluorescence microscope (Nikon, AX NIS-Elements 5.4, Nikon Corporation, Tokyo, Japan).

### 4.9. RT-qPCR Experiment

The young root, stem, leaf, flower, bud, and ovary from Yuzhi 11 were selected during the blooming flower periods. Additionally, we collected pericarp and seed tissue from plants that were pollinated after 6, 9, 15, and 28 d. The extraction of total RNAs, the synthesis of first-strand cDNA, and the RT-qPCR experiment were performed based on the standard method described in an existing study [24]. The endogenous *Siβ-tubulin* gene was used to normalize the expression level. Primer pairs for *SiGIF1* and *Siβ-tubulin* are shown in Supplementary Table S1. Relative expression levels were subsequently calculated by using the 2^−ΔΔCt^ method. All experiments were performed with three technical replicates and three biological replicates.

### 4.10. GIF Gene Family Homolog Detection in Sesamum and Phylogenetic Analyses

To obtain accurate *GIF* gene family sequences, we first downloaded the *GIF* family sequences of *Arabidopsis* (accession numbers: Q8L8A5, Q9MAL9, and Q93VH6) and rice (accession numbers: XP_015615240.1, XP_015618329.1, and XM_015774198.2) from the National Center for Biotechnology Information database. BlastP was used to screen homolog sequences in *Sesamum*, and the protein sequence of *Arabidopsis GIFs* was used as a query, with the e-value set to 1 × 10^−5^. All homologous sequences were aligned, and neighbor-joining trees were constructed using MEGA5 with default parameters [37]. The *Sesamum* genomes are from an existing study conducted in our lab [33].

## Figures and Tables

**Table 1:** Information of screened variant loci.

Type	Chr	Pos	REF ^(1)^	ALT ^(2)^	Gene_Position	Gene
SNP	9	15914090	G	A	splice_donor_variant	Sindi_2109900
SNP	9	15604373	T	G	intron_variant	Sindi_2107100
SNP	9	14333366	A	G	upstream_gene_variant	Sindi_2095300
SNP	9	15107600	T	G	intergenic_region	Sindi_2102600-Sindi_2102700
SNP	9	15107598	T	G	intergenic_region	Sindi_2102600-Sindi_2102700
InDel	9	15107594	T	TAG	intergenic_region	Sindi_2102600-Sindi_2102700
InDel	9	16876339	T	TAA	intergenic_region	Sindi_2118400-Sindi_2118500

^(1)^ Reference genome (Yuzhi 11) base sequence; ^(2)^ mutated base sequence.

## Data Availability

The sequence of *SiGIF1* was submitted to GenBank under accession number OR260083. The original contributions presented in this study are in the article/Supplementary Materials, and further inquiries can be directed to the corresponding authors.

## Supplementary Materials

The following supporting information can be downloaded at: https://www.mdpi.com/article/10.3390/plants13233294/s1, Figure S1 Protein sequence alignment of GIF1 from Yuzhi 11, rice and Arabidopsis, generated using MEGA 6.0 and visualized with DNAMAN software. Amino acids shaded in dark blue and blue represent 100% and >50% amino acid similarity, respectively; Table S1: Sequence of primers; Table S2: The quality of genome resequencing for F_2_ population; Table S3: Variant information of significant SNP loci and candidate variants screened in germplasm accessions; Table S4: Variant information of significant InDel loci and candidate variants screened in germplasm accessions; Table S5: Amplification validation of G15914090A SNP marker in population; Table S6: The protein sequence of SiGIF1 and Sigif1.
