# Supplementary material for: An Ethyl Methanesulfonate-Induced GIF1 Splicing Site Mutation in Sesame Is Associated with Floral Malformation and Small Seed Size
Source: Plants (Basel). 2024 Nov 23;13(23):3294. doi: 10.3390/plants13233294 (PMC11644617; doi:10.3390/plants13233294)
Supplement: Supplementary file 1 [file plants-13-03294-s001.zip › Supplementary figure.pdf]

```
Yuzhi_11-GIF1         MQQHLMQMQP.MMAAYY.PPNVTTDHIQQYLDENKSLILKIVESQNSGKLSECAENQAR   57
rice-GIF1            MQQQHLMQMNQGMMGGYASPTTVTTDLIQQYLDENKQLILAILDNQNNGKVEECARNQAK   60
Arabidopsis-GIF1      MQQHLMQMQP.MMAGYY.PSNVTSDHIQQYLDENKSLILKIVESQNSGKLSECAENQAR   57
Consensus              qqhl mqm  mm y  p  vt d iqqyldenk lil i   qn gk  eca nqa

Yuzhi_11-GIF1        LQRNLMYLAAIADSQPQPPTMHSQY..AAGGILQP..GAHYLQHQQA...QQMTPQSLMA   110
rice-GIF1            LQHNLMYLAAIADSQPPQTAAMSQY..PSNLMMQS..GARYMPQQSA...QMMAPQSLMA   113
Arabidopsis-GIF1     LQRNLMYLAAIADSQPQPPSVHSQYGSAGGGMIQGEGGSHYLQQQQATQQQQMTQQSLMA   117
Consensus            lq nlmylaaiadsqp      sqy          q    g  y    q a   q m  qslma

Yuzhi_11-GIF1        ARSSMLYG.....TQPYPSLQQQQALHSQISMSSGG..SGGLLLQGEPHSAGGSGS....   159
rice-GIF1            ARSSMMYAQPALSPLQQQQQQAAAAHGQLGMGSGGTTSGFSILHGEASMGGGGGGGAG   173
Arabidopsis-GIF1     ARSSMLYAQQQQQQQPYATLQHQQLHHSQLGMSSS...SGGGGSSG.LHILQG.......   166
Consensus            arssm y              q      h q   ms      sg       g       g

Yuzhi_11-GIF1        ...LGGGGFPDFVRSTPGDGLQTSSRTMASGSKQDMGSNVSAEGRGGS...DGGETLYLK   213
rice-GIF1            NSMMNAGVFSDFGRGGGGGG.............KEGSTSLSVDVRGANSGAQSGDGEYLK   220
Arabidopsis-GIF1     ....EAGGFHDFGRGKP.................EMGSGGGGEGRGGSS.GDGGETLYLK   204
Consensus                  g f  df  r                                rg        g   ylk

Yuzhi_11-GIF1        SAEDGN                                                         219
rice-GIF1            GTEEEG                                                         226
Arabidopsis-GIF1     SSDDGN                                                         210
Consensus
```

Supplementary Figure S1. Protein sequence alignment of GIF1 from Yuzhi 11, rice and Arabidopsis, generated using MEGA 6.0 and visualized with DNAMAN software. Amino acids shaded in dark blue and blue represent 100% and >50% amino acid similarity, respectively.
